# White matter changes after Gamma Knife Capsulotomy in patients with intractable obsessive-compulsive disorder

**DOI:** 10.1016/j.heliyon.2024.e34699

**Published:** 2024-07-18

**Authors:** Giorgio Spatola, Paul Triebkorn, Raphaelle Richieri, Christelle Baunez, Jean Farisse, Axelle Cretol, Eric Guedj, Viktor Jirsa, Jean Regis

**Affiliations:** a Fondazione Poliambulanza Istituto Ospedaliero, Department of Neurosurgery, Brescia, Italy; b Institut de Neurosciences des Systèmes (INS), UMR1106, Aix-Marseille Université, Marseilles, France; c Université Aix-Marseille, Marseille, France; d Aix Marseille Univ, CNRS, Centrale Marseille, Institut Fresnel, Marseille, France; e Department of Psychiatry, Sainte-Marguerite University Hospital, APHM, Hôpital de la Timone, France; f Institut de Neurosciences de La Timone, UMR 7289 CNRS & Aix-Marseille Université, 13005, Marseille, France; g AP-HM, Department of Functional and Stereotactic Neurosurgery, 13005, Marseille, France; h Département de Médecine Nucléaire, Aix Marseille Univ, APHM, CNRS, Centrale Marseille, Institut Fresnel, Hôpital de La Timone, CERIMED, Marseille, France

**Keywords:** Gamma Knife, Obsessive compulsive disorder, Y-BOCS, Diffusion weighted imaging, Fixel based analysis, Anterior limb of internal capsule

## Abstract

**Background:**

Anterior capsulotomy is one of the therapeutic options for refractory obsessive–compulsive disorder (OCD). Safety and efficacy of Gamma Knife Capsulotomy (GKC) have been demonstrated in the past.

**Objective:**

To characterize changes induced by GKC using a fixel-based analysis (FBA) and possible predictors of efficacy.

**Methods:**

Patients with OCD refractory to other therapies underwent bilateral GKC with 120 Gy as a maximum dose on the anterior limb of the internal capsule (ALIC). The clinical outcome was percent reduction in Yale- Brown Obsessive–Compulsive Scale (Y-BOCS). White Matter changes were analyzed using fixel-based analysis (FBA) for fibre density (FD), fibre-bundle cross-section (FC) and the combination of the two (FDC).

**Results:**

Seven patients underwent GKC. Median follow-up was 13 months (range 12–58 months). Mean (±SD) decrease in Y-BOCS score at last follow-up was 61 % ± 35 % with five patients considered as responders. FBA showed a symmetric FD reduction in the ALIC with extension to the anterior fronto-thalamic radiation; a reduction of FC along the superior longitudinal fasciculus (SLF) in both hemispheres with a predominance in the left one. Reductions in FDC were detected predominantly in the right hemisphere, with a similar pattern to FD reductions and associated with a positive correlation (p < 0.05) between Y-BOCS reduction and fibres passing in the ventral part.

**Conclusions:**

GKC is safe and efficient in reducing OCD severity in selected patients. Changes induced in white matter by GKC extend over the ALIC. Reduction of fibres passing the ventral part of the right sided ALIC correlates with better results.

## Graphical abstract

**Figure:**
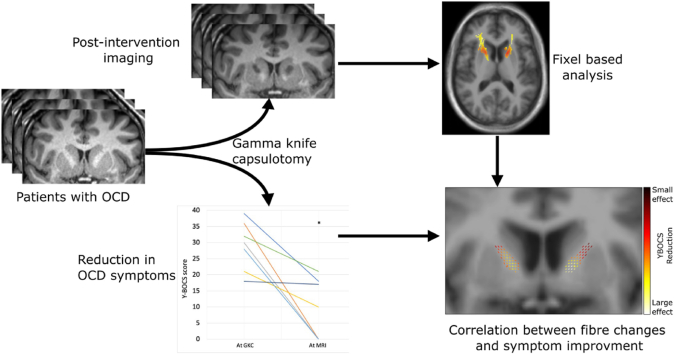

## 1 Introduction

Obsessive-Compulsive Disorder (OCD) is a mental health condition characterized by time-consuming and unwanted thoughts (obsessions) combined with repetitive behaviors or mental acts (compulsions) causing intense anxiety and distress. Treatment usually involves behavioral therapy, medication, or a combination often providing relief [1]. Unfortunately approximately from 30 to 50 % of individuals do not respond sufficiently to these interventions, leading to consideration of neurosurgical approaches in specific cases [[2], [3], [4]]. Pathophysiology of OCD involves dysregulation in corticostriatal-basal ganglia-cortical circuits, connecting orbitofrontal cortex (OFC), anterior cingulate, basal ganglia, and thalamus [5]. Ablative intervention (LITT, radiofrequency, focused ultrasound and Gamma Knife) and deep brain stimulation have been proposed for treatment refractory OCD [[6], [7], [8], [9], [10]]. Among them, Gamma Knife capsulotomy (GKC) and focused ultrasound create precise lesions without making any surgical incision or physically penetrating the brain and commonly targets the anterior limb of the internal capsule (ALIC) bilaterally in OCD. GKC side effects are rare and can manifest as transient headache and fatigue in the immediate post-procedure period while memory deficits and brain edema are possible but rare late side effects [4,11,12]. Recent advancements offer distinct advantages in terms of safety and comparative efficacy when compared to conventional methods. In OCD, some authors proposed to target more on the ventral part of the ALIC in the brain [8] and lowering the maximal dose from 180 Gy to 120 Gy [13]. As a whole, GKC yielded treatment efficacy of 40 %–80 % in uncontrolled trials [14].

Neuroimaging studies have investigated alterations in the white matter caused by neurosurgical interventions among OCD patients, employing diffusion-weighted imaging (DWI) and tractography. However, these studies often used pre-intervention imaging only or average connectomes derived from healthy subjects, limiting their ability to identify potential predictive fiber bundles correlated with clinical improvement [[15], [16], [17], [18]]. Only one study has compared DWI data within the same patient group before and after radiofrequency ablation, revealing changes in fractional anisotropy (FA) along the anterior thalamic radiation and associations with clinical outcomes [19].

Traditionally, white matter integrity has been analyzed using the diffusion tensor model (DTI), which fits a tensor to the diffusion signal in each voxel of the white matter. The disadvantage of this approach however is that DTI cannot account for multiple crossing fibres within a voxel, which may lead to overlooking or wrong interpretation of changes [20,21]. More recent approaches, such as constrained spherical deconvolution, can identify multiple fibres within a voxel [22], which enables fixel based analysis (FBA) to test for fibre specific changes in white matter [23]. In a voxel with crossing fibres multiple fixels (i.e. fibre elements) may be identified and statistical tests can be performed separately on each. This framework allows one to compare fibre density (FD), fibre-bundle cross-section (FC) and the combination of the two fibre density and bundle cross-section (FDC), which are metrics for the intra-axonal volume of each fixel, the spatial extent of a macroscopic fibre pathway and the total intra-axonal volume within a pathway, respectively [23]. The approach has previously been used to study fibre specific white matter changes in Alzheimer [21], Parkinson [24], motor neuron disease [25] and temporal lobe epilepsy [23].

This study demonstrates for the first time the effect of GKC on white matter using pre- and post-GKC diffusion imaging of the same patients. By utilizing FBA, we uncover extended global microstructural changes in white matter, helping to improve our understanding of the pathophysiology of the disease. A significant scientific gap remains our limited understanding of the precise target within the ALIC necessary for clinical improvement in OCD. By correlating GKC-induced white matter changes with observed clinical outcomes, we aim to improve future treatment strategies for OCD.

## 2 Methods

We included all patients who underwent GKC for intractable OCD from 2017 to 2021 at La Timone Hospital in Marseille. Patients with less than 1 year of follow-up and without a preoperative DTI imaging were excluded. Consent for chart review was obtained from all patients and authorization for data management was obtained from the local ethical committee.

The study was approved by the institutional board and the ethical board of the College de neurochirurgie IRB #1 : 2023/09 with the n° IRB00011687.

### 2.1 Patient selection

Assessment for suitability for radiosurgery was conducted by a committee comprising members from neurosurgery, neuropsychology and psychiatry. This committee reviewed all available medical, psychiatric, and neuropsychological records [2]. The patient's informed consent, along with that of their legal representative if required, was obtained before the procedure. They presented a primary diagnosis of OCD for a minimum of 5 years without any remission periods assessed by at least two psychiatrists and classified as unresponsive to all available treatments. The patient must have severe OCD, which is significantly impairing their ability to function in daily life. Additional eligibility criteria included a baseline Y-BOCS score ≥24 or a subscore for obsession or compulsion ≥18. These scores had to remain consistent for at least 12 months prior to GKC. Patients with Y-BOCS score between 20 and 24, with attempts to suicide or important pharmacological side effects were carefully evaluated and included only if the committee couldn't proprose any other solution. Patients were excluded if they presented a mental retardation, any medical/neurologic contraindication to GKC or a pre-treatment of neuroimaging with structural abnormalities.

### 2.2 Outcome measures

Assessment instruments included the Structured Clinical Interview for DSM-V Axis I Disorders, the Yale-Brown Obsessive-Compulsive Scale (Y-BOCS), the Quick Inventory depression scale- Self report (QIDS-SR16), the Clinical Global Impression-Severity (CGI-S) and Improvement (CGI-I) and the Global Assessment of Functioning scales (GAF).

### 2.3 Radiosurgical procedure

All patients were admitted to the hospital the day prior to treatment. Frame was fixed under local anesthesia and Radiosurgery was administered using the Elekta Gamma Knife Unit Perfexion. GKC was performed using the same technique as previously published. The maximum prescription dose was 120 Gy [13]. Patients were discharged the day after treatment after a general neurological examination. Subsequent to GKC, patients received ongoing monitoring from their attending psychiatrist and general practitioner.

### 2.4 Clinical follow-up

Assessments were conducted at the neurosurgical clinic at La Timone Hospital. Functional neuroradiological follow-up examinations were scheduled at 1 year, and then annually up to the fifth year post-GKC. Y-BOCS, GAF, QIDS-SR and GCI-I scales were administered at outpatient visits by the psychiatrist and psychologists. Patients-with- ≥35%- decrease-in Y-BOCS score and a CGI-I rating of 1 (“very much improved”) or 2 (“much improved”) at last follow-up were classified as responders.

### 2.5 Image acquisition and processing

Pre- and 1-year post-operative T1 weighted (MPRAGE sequence, 2.01 ms/2.3s (TE/TR), 1x1x1mm^3^ voxel size) and DWI (84 ms/7.3–9.6s (TE/TR), voxel size 2.2x2.2 × 2.2 mm^3^, bval = [0,1000], 64 directions, AP phase encoding) MRI data were acquired for each patient. For two patients the 1-year post-operative data was not available and instead the 2- and 4-year follow up data was used, respectively. The T1 weighted images were processed using the Freesurfer software package [26] and a lesion mask was segmented manually on the post-operative image. Diffusion weighted images were processed using the MRtrix3 software package [22]. The DWI data was corrected for noise [27], Gibbs ringing artifacts [28], movement, epi- and eddy current distortions [29]. Because the diffusion data was acquired in anterior to posterior phase encoding direction only, we used Synb0 DisCo [30] to estimate a synthetic undistorted b0 image that was used for distortion correction. Response functions were estimated for gray matter, white matter and cerebrospinal fluid diffusion signals [31] and averaged across subjects. Diffusion images were upsampled to 1.25 mm^3^ isotropic voxel size for improved anatomical contrast and image alignment [32]. Subsequently fibre orientation distributions (FOD) were estimated using single-shell multi-tissue constrained spherical deconvolution [33] and were corrected for intensity inhomogeneities [34]. To align images across subjects we constructed a population template FOD image. First, an intra-subject template was created by rigid registration of pre- and post-GKC imaging data. Second, an inter-subject template was created by affine and non-linear registration of the intra-subject templates. Each subject's FOD image was then nonlinearly registered to the inter-subject template. The same transformation was used to align all T1 weighted images, to obtain an average structural template image. This structural template was processed with Freesurfers recon-all pipeline to obtain a 5-tissue-type image for anatomically constrained tractography [35]. A probabilistic tractogram with 20 million tracts was generated from the template FOD image, which was subsequently downsampled to 2 million tracts using SIFT [36].

### 2.6 Fixel-based analysis

The template FOD image was used to compute a fixel mask and fixel based metrics, i.e. FD, FC and FDC, were extracted for each subject following the approach detailed in Ref. [23].

**Fig. 1:**
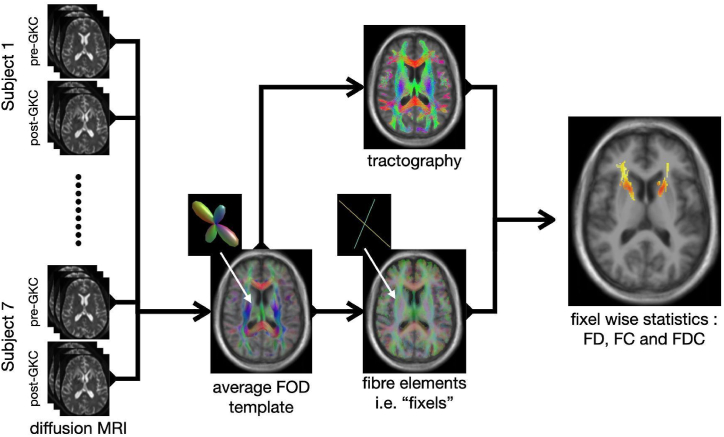
FBA Workflow overview. Subject wise pre- and post-GKC diffusion weighted MRI data were processed and registered to create a FOD template image. The FODs of the template were used to create a fixel mask and to run tractography for connectivity-based fixel enhancement. Fixel based metrics (i.e. FD, FC and FDC) were computed for each subject and their difference from pre-to post-GKC was tested for statistical significance.

Connectivity-based smoothing of the fixel metrics was performed with a 10 mm FWHM Gaussian kernel using the fixel-to-fixel connectivity as estimated from the template tractogram. For statistical analysis we used connectivity-based fixel enhancement [25] with non-parametric permutation testing to obtain family wise error (FWE) corrected p-values [37]. The general linear model (GLM) tested the difference in fixel metrics from pre-to post-GK intervention. For FC and FDC we used the intracranial volume estimate from recon-all as a nuisance regressors [38]. Additionally, we performed a region of interest analysis focusing only on the ALIC, delineated by a manually drawn mask, in which we investigated the relation between change in fixel metrics and the change in YBOCS score. A threshold FWE-corrected p-value = 0.05, was used to distinguish statistically significant changes in fixels. Fig. 1 shows an overview of the FBA workflow.

### 2.7 Statistical analysis

Descriptive statistics for the continuous variables were reported as mean, standard deviation -SD-, median and range. Longitudinal evaluations (pre-post intervention) for continuous variables were calculated using the Wilcoxon test for paired non-normally distributed samples. Generalized estimating equation models were used to find out any predictors of the changes of the main clinical outcomes. Significance level was set at p = 0.05. All analyses were performed using the R statistical software (version 4.3.2, R Core Team 2023; https://www.R-project.org/).

## 3 Results

### 3.1 Demographics and clinical evaluation

All clinical data and descriptive statistics are summarized in Table 1. Seven patients were finally included in this sample. The median age at treatment was 42 years (range 31–61, mean 43.1 ± 11.4). The median temporal interval between the diagnosis and GKC was 24 years (range 7–40, mean 26.3 ± 11.2). Before GKC, the median Y-BOCS score was 30 (range 18–39, mean 29.1 ± 7.6) with a median obsession score of 16 (range 11–20, mean 15.9 ± 3.5) and compulsion score of 16 (range 0–19, mean 13.2 ± 6.7.9 ± 8).

**Table 1:** Descriptive statistics with YBOCS baseline and FU.

	Sub01	Sub02	Sub03	Sub04	Sub05	Sub06	Sub07	Mean	Std	Median
Years of OCD	24	32	37	21	40	23	7	26.3	11.2	24
AGE	51	42	61	36	50	31	31	43.1	11.4	42
Follow up (months)	16	12	58	13	24	12	13	21.0	16.9	13
Left lesion [mm3]	197	42	45	166	37	50	360	128.1	121.6	50
Right lesion [mm3]	98	26	30	118	6	52	276	86.6	92.7	52
Total volume lesion	295	68	75	284	43	102	636	214.7	212.9	102
Y-BOCS preOP	39	36	30	21	28	32	18	29.1	7.6	30
Obsessions preOp/20	20	19	15	12	11	16	18	15.9	3.4	16
Compulsions preOp/20	19	17	15	9	17	16	0	13.2	6.7	16
Y-BOCS postOP	18	0	0	10	0	21	17	9.4	9.4	10
Obsession PostOP	9	0	0	4	0	11	17	5.9	6.7	4
Compulsion POstop	9	0	0	6	0	10	0	3.6	4.6	0
Y-BOCS reduction [%]	53.85	100	100	52.38	100	34.37	5.56	63.7	37.5	53.85
Response	YES	YES	YES	YES	YES	NO	NO			
CGI-S preOP	7	7	6	5	5	7	5	6.0	1.0	6
CGI-I postOP	2	4	6	4	2	1	4	3.3	1.7	4
QIDS-SR preOP	13	13	13	10	14	20	5	12.6	4.5	13
QIDS-SR postOP	5	0	19	13	2	0	4	6.1	7.2	4
GAF preOP	39	38	47	55	45	33	53	44.3	8.1	45
GAF postOP	45	90	40	55	72	70	53	60.7	17.5	55

**Fig. 2:**
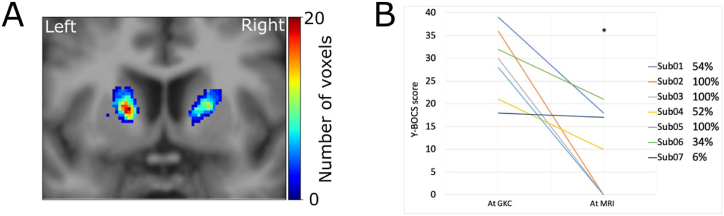
(A) Coronal section of the template T1w image with manually segmented lesion masks summed across the anterior-posterior direction and displayed as a heatmap. (B) Change in Y-BOCS score for each subject from pre-post-GKC (*p = 0.02) and percentage reduction.

In median post-op MRI was performed 13 months after GKC (range 12–58 months, mean 21 ± 16.9). At that moment five patients (71 %) achieved a full response. Two patients were considered partial- and non-responder, respectively. The size of the lesion resulting from GKC was assessed for all patients in both hemispheres at this point in time (Fig. 2A). Left and right hemispheric median lesion volume was 50 mm^3^ (range 37–360, mean 128.1 ± 121.6) and 52 mm^3^ (range 6–276, mean 86.6 ± 92.7), respectively. No statistical differences in lesion volumes were detected between responders and non responders and no correlation was found with Y-BOCS reduction.

The median pre-GKC Y-BOCS score of 30 (symptoms rated as severe) showed a statistically significant decrease (p = 0.02) to a median value of 10 (range 0–21, mean 9.4 ± 9.4) and classified as mild (Fig. 2B). CGI-S was in median 6 at preop evaluation and GCI-I at postop evaluation reached a median of value of 4. Depressive symptoms at MRI decreased at a median value of 4 (QIDS range 0–19 mean 6.14 ± 7.2) with no statistically significant difference (p = 0.16). The GAF at MRI increases to a median value of 55 (range 40–90, mean 60.7 ± 17.5) without reaching a statistically significant difference (p = 0.18). All potential factors concerning the patient (such as age, duration of OCD symptoms, pre-existing obsessions and compulsions, duration of FU) as well as imaging-related aspects (lesion volumes) have been considered in order to categorize potential predictive elements but none of them seems to be predictive of a better result. No adverse clinical or radiological side effects have been reported; neuropsychological testing of patients demonstrated the stability of all motor and cognitive functions.

### 3.2 Fixel based analysis pre-to post-GK

**Fig. 3:**
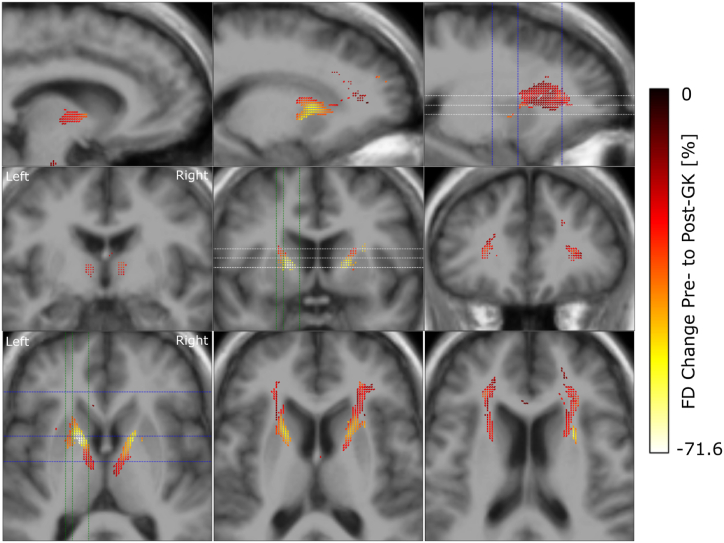
Reduction in fibre density from pre-to post-GK capsulotomy. From the top to the bottom we display one double and two single rows of sagittal, coronal and axial slices of the template T1w image, with overlay of color coded fixels. Color indicates the change from pre-to post-GK in percent. Only fixels with a FWE-corrected p-value <0.05 are displayed. White, blue and green dashed lines indicate the positions of the axial, coronal and sagittal slices, respectively.

**Fig. 4:**
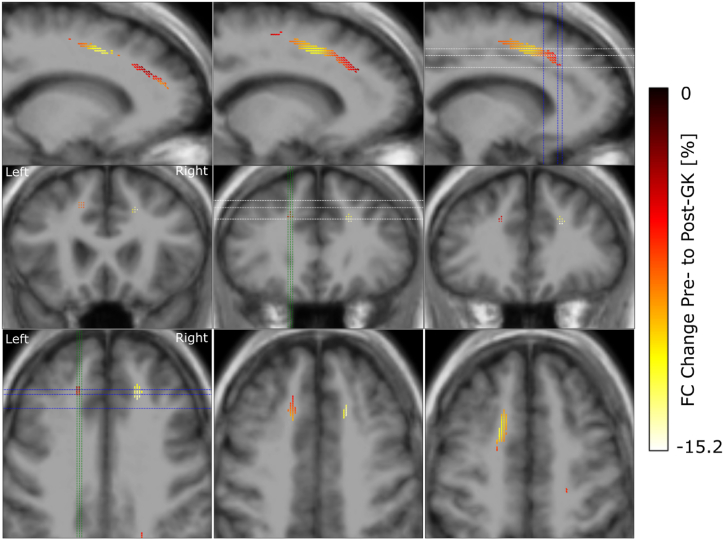
Reduction in fibre cross-section from pre-to post-GK capsulotomy. From the top to the bottom we display rows of sagittal, coronal and axial slices of the template T1w image, with color coded fixels on top. Color indicates the change from pre-to post-GK in percent. Only fixels with a FWE-corrected p-value <0.05 are displayed. White, blue and green dashed lines indicate the positions of the axial, coronal and sagittal slices, respectively.

**Fig. 5:**
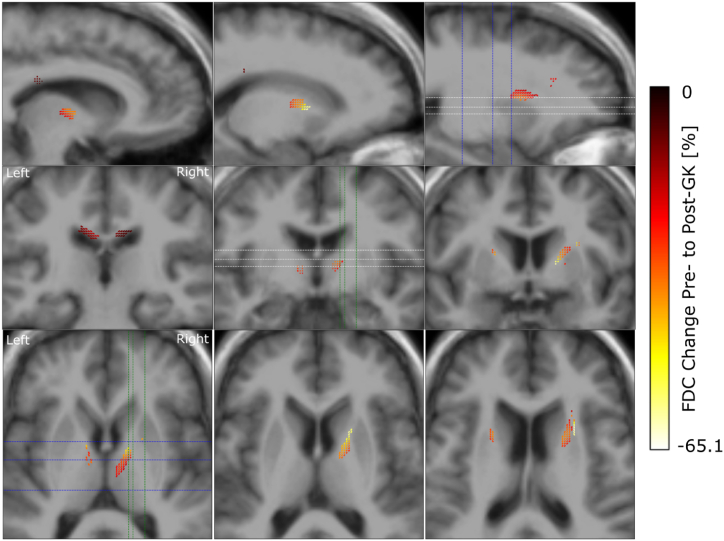
Reduction in the combined measure of fibre density and cross-section from pre-to post-GK capsulotomy. From the top to the bottom we display rows of sagittal, coronal and axial slices of the template T1w image, with color coded fixels on top. Color indicates the change from pre-to post-GK in percent. Only fixels with a FWE-corrected p-value <0.05 are displayed. White, blue and green dashed lines indicate the positions of the axial, coronal and sagittal slices, respectively.

After preprocessing of the imaging data we used FBA to investigate the change in FD, FC and FDC caused by GKC in 7 patients (Fig. 1). We observed a symmetric FD reduction across hemispheres with largest effect sizes in the ALIC, close to the location of the GK lesions (Fig. 3). The effect however is not purely localized in the ALIC but extends along the pathway of the anterior thalamic radiation. A reduction of FC was seen along the SLF of the frontal cortex, extending along the anterior to posterior axis (Fig. 4). This reduction was observed in both hemispheres with a stronger and more extended effect on the left. Reductions in FDC were detected predominantly in the right hemisphere, with a more dorsal extent than FD reduction (Fig. 5).

### 3.3 Connectome analysis

**Fig. 6:**
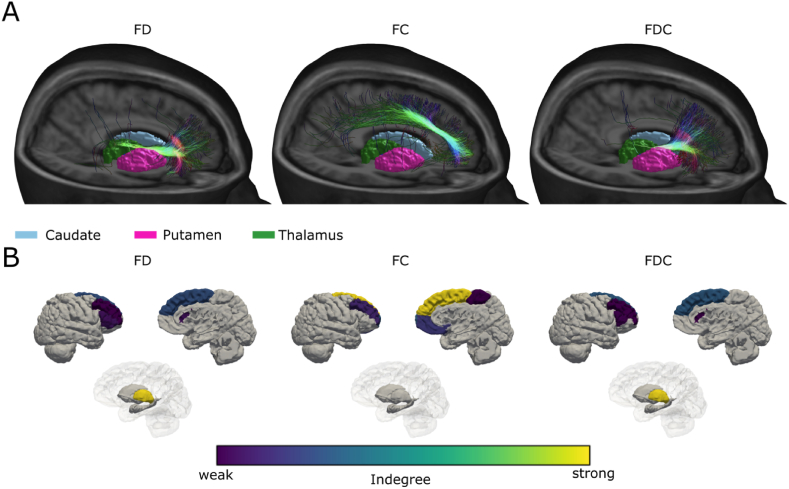
(A) Volume rendering of the T1w template image, sectioned on the right-hemisphere, with meshes of subcortical structures, to display the location of fibre pathways affected by GK capsulotomy for FD, FC and FDC. Fibres were filtered by the amount of significant fixels they were passing. (B) 3D surface renderings of the right-hemispheric cortex (lateral and mesial view) and subcortex (medial view) for connectome analysis of fibres affected by GK capsulotomy for FD, FC and FDC. Colors indicate the degree (thresholded at the 90th percentile) of each brain area, connected by the affected fibres. Each fibre was weighted by the amount of significant fixels it were passing.

To further investigate the affected connections and involved brain areas, fixel statistics were mapped onto the tracts passing through them. Subsequently tracts were assigned to regions of the Desikan-Killiany atlas [39], to construct a connectome in which each tract was weighted by the amount of significant fixels it would pass through. Fig. 6A shows 3D renderings of the affected fibres and their anatomical location. Fibres with reduced FD mainly connect the Medial-dorsal-Thalamus (MDT) with the PFC. Fibres with reduced FC extend along the SLF of the frontal white matter, connecting prefrontal, frontal and central cortex. Fibres affected by FDC reduction show a similar pattern to FD, however the fibres passing through the ALIC extend to more dorsal parts of the PFC. Fig. 6 B displays the cortical and subcortical regions of the right hemisphere which are connected by effected fibres. Changes in FD are mainly observed in connections from the anterior part of thalamus to the superior frontal gyrus (SFG), rostral middle frontal gyrus (rMFG) and pars-orbitalis and -triangularis of the inferior frontal gyrus. Connections affected by changes in FC localize between SFG, rMFG, medial OFC, as well as extend towards the paracentral cortex. Connections affected by changes in FDC extend from the Thalamus to the SFG, rMFG and pars-triangularis.

### 3.4 FBA in relation to YBOCS

**Fig. 7:**
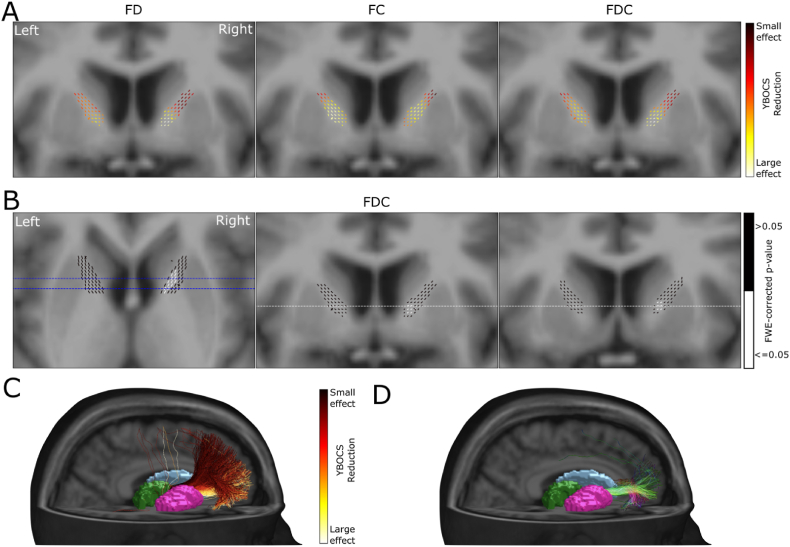
Relation of reduction in fixel metric and reduction in YBOCS score within the ALIC. (A) Coronal sections of the template T1w image, with overlaid color coded fixels of effect sizes for FD, FC and FDC in relation to YBOCS reduction. (B) Axial and coronal sections of the template T1w images, with overlaid color coded fixels. White fixels show a significant linear relation between reduction in FDC and reduction in YBOCS. (C) Volume rendering of the T1w template image, sectioned on the right-hemisphere, with meshes of subcortical structures. Displayed fibres are passing from the Thalamus through the ALIC and are colored by the average effect size of YBOCS reduction explained by FDC change from panel (A). (D) Fibres passing from the thalamus through the significant fixels of the ALIC from panel (B).

We investigated the relation of reduction in fixel metrics with the reduction of YBOCS score by including the percentage change of YBOCS as a regressor into the GLM. As the ALIC is the target of GKC, we chose a region of interest approach to increase statistical power and only analyzed fixels within the ALIC. Fig. 7A depicts the absolute effect sizes of changes in YBOCS scores explaining changes in fixel metrics. For all 3 metrics higher effect sizes are displayed in ventral parts of the ALIC. However only for FDC, these effects were significant (Fig. 7B). When mapping the effect size of YBOCS reduction due to FDC change onto the fibres passing the ALIC, a clear ventral-dorsal gradient is visible (Fig. 7C). Fibres passing through the ventral part of the ALIC are more associated with YBOCS reduction than those passing through the dorsal parts. Finally when displaying only those fibres passing through significant fixels, we see that they are connecting the MDT with the ventral PFC (Fig. 7D).

## 4 Discussion

This is the first study using FBA, as a method for fiber specific analysis of white matter changes, to compare pre- and post-GKC diffusion weighted MRI in patients with refractory OCD. Our results show significant changes in white matter integrity involving connections from the PFC towards the MDT and anterior thalamus as well as changes in the superior longitudinal fasciculus. Furthermore, we found a significant correlation between Y-BOCS improvement and changes in white matter of the ventral part of the ALIC, possibly identifying a more effective target for future GKC.

In a recent metanalysis, the reduction can reach 50.4 % (±22.7 %) for ablative techniques and 40.9 % (±13.7 %) for DBS [7]. Our data are in line with these results. No clinical or radiological adverse effects were reported during the FU period, confirming the efficacy and safety of treating patients with a maximal dose of 120 Gy [13].

### 4.1 FBA results in relation to anatomy and pathophysiology of OCD

We found significant changes in white matter structure after GKC. The most expected result was the change in FD along the anterior thalamic radiation, a fibre bundle that has previously been shown to be implicated with capsulotomy in LITT [9], GK [19] and radiofrequency ablation [40]. FD reduction in this study is related to Wallerian degeneration of axons after lesioning the ALIC. The reduction was stronger within the ALIC and decreased along the thalamic radiation towards the PFC and the Thalamus. Mapped onto cortical parcellations, these fibres connect the Thalamus with regions of the prefrontal and OFC. Activity changes of these areas have previously been shown to be related to OCD [41]. In this study, we describe a decrease in FC along the SLF, extending along the anterior to posterior axis in both hemispheres, with a more pronounced and widespread effect noted in the left hemisphere but no correlation to Y-BOCS reduction. Changes in FC affected the SLF with a reduction of at most 15 %. Decreasing cross section is related to atrophy of a fibre bundle with subsequent shrinkage of the white matter tissue. While this is a new finding in a GKC series, the significance of the SLF has already been demonstrated in patients with OCD together with the cingulum in cingulo-opercular network that might underlie observations of inhibitory control in OCD [41]. In a previous study, OCD patients exhibited significantly lower FA and altered probability density distribution along various brain regions, including the corpus callosum, cingulum, SLF and inferior fronto-occipital fasciculus bilaterally [42]. Some authors further emphasized the correlation between the severity of OCD and white matter alterations in distinct brain regions including the left and right SLF, among others [43]. The FDC measures the overall intra-axonal volume of a fibre bundle. It showed changes along the anterior thalamic radiation with a higher effect on the right hemisphere. Compared to the FD changes, the affected fixel tended to extend more into the dorsal parts of the PFC. This is probably due to the combination of FD and FC, with the FC change happening in the dorsal PFC too.

### 4.2 FBA results and correlation with Y-BOCS reduction

Our results indicate that higher effect sizes were observed in ventral parts of the ALIC for all three metrics, but only the effect sizes for FDC in the right ventral part of the ALIC were found to be significantly associated with Y-BOCS reduction. Here the involved fibres are part of the anterior fronto-thalamic radiation connecting the thalamus to pars orbitalis and SFG. The relevance of the right hemisphere in GKC for OCD had already been supported in a prior study. Authors found that all post GKC MRI showing changes in the ventral part of the right ALIC correlated with better clinical results [12].

Only two studies have previously focused attention on fibre bundles and clinical results in patients treated with GKC. The first study observed changes in the medial and lateral fibre pathways originating from the OFC in patients with OCD compared to patients with Parkinsons and morbid obesity [15]. In the other study Bouwens et al. analyzed fibres passing the ALIC using a normative connectome. They found a trend towards fibres passing through the ventral part of the ALIC leading to a larger reduction in Y-BOCS scores, yet without statistical significance [16]. This is in line with a study analyzing changes after LITT capsulotomy, which found that responder patients were associated with an ablation extending towards the ventral part of the ALIC, affecting fibres mainly connecting to the OFC instead of the dorsal PFC. Using DBS and a normative tractography it was shown that the connectivity of stimulation sites to the MFG correlated with Y-BOCS improvement [17]. Patients undergoing ALIC radiofrequency ablation with a high pre-operative connectivity between thalamus and dorsolateral PFC or anterior cingulate cortex would have larger Y-BOCS reduction [40]. Same results have been shown using a normative connectome after LITT capsulotomy. In their series Satzler et al. describe how voxels linked to responder status were primarily situated in the ventral ALIC with the most prominent responder streamlines located in a more ventral position and extended into the frontal lobe [9]. In the only other study with a comparison between pre and post DWI after thermocapsulotomy a positive correlation was observed between pre-surgical FA in the left LFC, the right cingulum and change in Y-BOCS score, but no correlation was found between FA changes and patient improvement [19]. Differently, examining optimal connectivity through the connectome in patients treated with DBS revealed that the extent of connectivity between stimulation sites and the medial and lateral PFC was a significant predictor of clinical improvement [17]. While all these studies affirm the significance of disrupting or modulating the anterior fronto-thalamic radiation there are some incongruences of which specific fibres can be real predictors. This could be due to variations in surgical techniques employed for capsulotomy or stimulation, different methodologies in analyzing white matter changes on DTI or the utilization of unified connectomics, interindividual variances in fibre distribution within the ALIC.

## 5 Limitations

The small size of our sample represents the major limitation that poses a challenge in drawing robust conclusions. Employing longitudinal analysis on the DWI of the same patients may be helpful in enhancing the reliability of the results. GKC unlike other methods takes time to achieve Y-BOCS improvement and repeating the evaluation with longer follow-up could help in being more accurate in finding predictors.

## 6 Conclusions

GKC is safe and effective in reducing OCD severity in selected patients. FBA offers a more nuanced and detailed approach to characterize white matter microstructure compared to traditional FA analysis. Changes induced in white matter by GKC extend over the ALIC. Fibres passing the ventral part of the right sided ALIC moving towards the pars orbitalis and the anterior part of SFG seem to correlate with better results. While these promising outcomes are noteworthy, additional investigations with extended follow-up periods are imperative to validate these findings.

## Funding

The research has received support from the European Union’s Horizon Europe Programme under the Specific Grant Agreement No. 101147319 (EBRAINS 2.0 Project) and No. 101137289 (Virtual Brain Twin Project), and 10.13039/501100001665Agence Nationale de la Recherche (ANR) under the France 2030 program, reference ANR-22-PESN-0012.

## Data availability statement

Has data associated with your study been deposited into a publicly available repository? - No.

Please select why. - The data that has been used is confidential.

## CRediT authorship contribution statement

**Giorgio Spatola:** Writing – original draft, Formal analysis, Data curation, Conceptualization. **Paul Triebkorn:** Writing – original draft, Software, Formal analysis, Conceptualization. **Raphaelle Richieri:** Writing – review & editing, Supervision, Data curation. **Christelle Baunez:** Writing – review & editing, Data curation. **Jean Farisse:** Supervision, Data curation. **Axelle Cretol:** Data curation. **Eric Guedj:** Writing – review & editing, Supervision. **Viktor Jirsa:** Writing – review & editing, Supervision, Funding acquisition. **Jean Regis:** Writing – review & editing, Validation, Supervision, Data curation.

## Declaration of competing interest

The authors declare the following financial interests/personal relationships which may be considered as potential competing interests:Jirsa Viktor reports financial support was provided by 10.13039/100007586Aix-Marseille University. If there are other authors, they declare that they have no known competing financial interests or personal relationships that could have appeared to influence the work reported in this paper.
